# Short term *Candida albicans *colonization reduces *Pseudomonas aeruginosa*-related lung injury and bacterial burden in a murine model

**DOI:** 10.1186/cc10276

**Published:** 2011-06-20

**Authors:** Florence Ader, Samir Jawhara, Saad Nseir, Eric Kipnis, Karine Faure, Fanny Vuotto, Chanez Chemani, Boualem Sendid, Daniel Poulain, Benoit Guery

**Affiliations:** 1 Department of Infectious Diseases, Croix-Rousse Hospital, 104 Grande-Rue de la Croix-Rousse, Lyon, F-69004, France; 2 Inserm U851 Finovi Centre d'Infectiologie, Claude Bernard Lyon 1 University, 321 avenue Jean Jaurès, Lyon, F-69007, France; 3 Inserm U799 Physiopathologie des Candidoses IFR 114, Lille II University School of Medicine and Lille University Hospital, 1 place de Verdun, 59037 Lille cedex, France; 4 Intensive Care Unit, Calmette Hospital, Lille University Hospital, boulevard du Pr Leclercq, 59037 Lille cedex, France; 5 EA 2689 IFR 114, Lille II University School of Medicine and Lille University Hospital, 1 place de Verdun, 59037 Lille cedex, France

## Abstract

**Introduction:**

*Pseudomonas aeruginosa *is a frequent cause of ventilator-acquired pneumonia (VAP). *Candida *tracheobronchial colonization is associated with higher rates of VAP related to *P. aeruginosa*. This study was designed to investigate whether prior short term *Candida albicans *airway colonization modulates the pathogenicity of *P. aeruginosa *in a murine model of pneumonia and to evaluate the effect of fungicidal drug caspofungin.

**Methods:**

BALB/c mice received a single or a combined intratracheal administration of *C. albicans *(1 × 10^5 ^CFU/mouse) and *P. aeruginosa *(1 × 10^7 ^CFU/mouse) at time 0 (T0) upon *C. albicans *colonization, and Day 2. To evaluate the effect of antifungal therapy, mice received caspofungin intraperitoneally daily, either from T0 or from Day 1 post-colonization. After sacrifice at Day 4, lungs were analyzed for histological scoring, measurement of endothelial injury, and quantification of live *P. aeruginosa *and *C. albicans*. Blood samples were cultured for dissemination.

**Results:**

A significant decrease in lung endothelial permeability, the amount of *P. aeruginosa*, and bronchiole inflammation was observed in case of prior *C. albicans *colonization. Mortality rate and bacterial dissemination were unchanged by prior *C. albicans *colonization. Caspofungin treatment from T0 (not from Day 1) increased their levels of endothelial permeability and lung *P. aeruginosa *load similarly to mice receiving *P. aeruginosa *alone.

**Conclusions:**

*P. aeruginosa*-induced lung injury is reduced when preceded by short term *C. albicans *airway colonization. Antifungal drug caspofungin reverses that effect when used from T0 and not from Day 1.

## Introduction

Ventilator-associated pneumonia (VAP) occurs in a considerable proportion of patients undergoing mechanical ventilation and is associated with substantial morbidity, a two-fold increase in mortality rate, and excess cost [1]. Tracheobronchial colonization (TBC) and duration of mechanical ventilation are the two most important risk factors for VAP [2,3]. *Pseudomonas aeruginosa *is one of the most frequent causative microorganisms of VAP [2-4]. Several studies have reported the presence of *Candida *species in the airway specimens of immunocompetent ventilated patients [5,6]. *Candida *TBC occurs in 17% to 28% of ICU patients receiving mechanical ventilation for more than 48 hours [7-9]. Although the relationship between tracheal biofilm and VAP is based on one small observational study, *P. aeruginosa *is the most common pathogen retrieved from endotracheal tube biofilm in patients with VAP [10]. *P. aeruginosa *and *C. albicans *coexist predominantly as biofilms rather than as free-floating (planktonic) cells on abiotic medical devices (catheters and prostheses) [11,12].

The question of their interplay has been addressed by several experimental and clinical studies. So far, *in vitro *studies suggest that the interaction between *C. albicans *and *P. aeruginosa *is likely to be antagonistic. When mixing *in vitro *cultures, *P. aeruginosa *is involved in killing *C. albicans *filaments associated with biofilm formation [13]. Additionally, quorum-sensing signaling molecules of *P. aeruginosa *impair *C. albicans *yeast-to-hyphae transition [14]. The relative *C. albicans *hyphal-binding affinity within biofilm is reported to be lower for *P. aeruginosa *than for *Staphylococcus aureus *[15]. In contrast, a synergistic relationship is described *in vivo *with a recent study showing that *C. albicans *TBC facilitates *P. aeruginosa *pneumonia occurrence in a rat model [16]. A recent clinical study suggested an interaction between *C. albicans *and *P. aeruginosa *[8]. The authors identified *Candida *spp. tracheobronchial colonization as an independent risk factor for *P. aeruginosa *pneumonia. No cause-and-effect relationship was demonstrated in that study. In addition, *Candida *spp. tracheobronchial colonization and *P. aeruginosa *pneumonia could both be a consequence of prior antibiotic treatment. Further, the median duration of mechanical ventilation in that study was 13 days. Therefore, the results could not be generalized to patients with shorter duration of mechanical ventilation. Another recent preliminary case-control study suggested that antifungal treatment might be associated with reduced risk for VAP or TBC related to *P. aeruginosa *[9], although no definite conclusion can be drawn from this observational retrospective single-center study including a small number of patients.

The study of *P. aeruginosa *and *C. albicans *interactions in the respiratory tract aims at more effectively understanding the balance between microbial ecology and bacteria-related pathogenesis. This issue has major environmental and medical consequences. The present study proposes to investigate *P. aeruginosa*-related lung injury in mice previously colonized with *C. albicans *and to evaluate the impact of caspofungin antifungal treatment.

## Materials and methods

### Animals

BALB/c mice (20 to 25 g) purchased from Charles River Laboratories (Domaine des Oncins, L'Arbresle, France) were housed in a pathogen-free unit of the Lille University Animal Care Facility and allowed food and water *ad lib*. All experiments were performed with the approval of the Lille Institutional Animal Care and Use Committee.

### Growth conditions for bacterial and yeast strains

The wild type strain *Pseudomonas aeruginosa *PAO1 was grown in Luria-Bertani medium at 37°C for 16 h and was centrifuged at 3,000 × g for 10 minutes. The bacterial pellets were washed twice and diluted in an isotonic saline solution to obtain an optical density of 0.63 to 0.65 nm determined by spectrophotometry [17].

The reference strain *C. albicans *SC5314 was maintained at 4°C on Sabouraud dextrose agar (SDA) [18]. For the study, cell of broth test isolates were grown in SDA at 37°C in a shaking incubator for 18 h.

### Mice infection

Mice were infected by direct intratracheal inoculation under short anaesthesia with inhaled sevoflurane (Servorane™, Abbott, Queenborough, UK) as previously described [17]. For each mouse, 50 μl of fungal or bacterial suspension containing 2 × 10^6 ^or 2 × 10^7 ^or 2 × 10^8 ^colony-forming units (CFU)/ml of yeasts or 2 × 10^8 ^CFU/ml of bacteria respectively, was instilled. Control mice received 50 μl of sterile saline solution.

### Treatment with caspofungin

Caspofungin (Merck & Co. Inc., Whitehouse Station, NJ, USA) was injected intraperitoneally once daily either from T0 or from 24 h post-*C. albicans *challenge. The full recommended dose of 1 mg/kg was administered the first day of treatment and then 0.8 mg/kg was administrated on Days 2, 3, and 4.

### Quantitative blood culture and pulmonary bacterial and fungal loads

For bacterial blood culture, 100 μl of blood was plated on bromocresol purple (BCP) agar plates for 24 h at 37°C to allow for *P. aeruginosa *growth. In co-infected groups, BCP agars were treated with 50 μg per plate of caspofungin. For fungal blood culture, the same amount was plated on yeast peptone dextrose (YPD) agar plates containing 1% yeast extract, 1% peptone, 2% D-glucose and 500 mg/l amikacin sulphate and incubated for 48 h at 37°C to allow for *C. albicans *growth.

For quantification of lung bacterial loads, lungs were removed after exsanguination *via *intracardiac puncture and homogenized in 0.9 ml of sterile isotonic saline solution. Viable bacteria were counted after serial dilutions of 100 μL of lung homogenate on BCP agar plates for 24 h at 37°C to allow for *P. aeruginosa *growth. Similarly, another 100 μL of lung homogenate was plated on YPD plates for 48 h to allow for *C. albicans *growth. In co-infected groups, agar was treated with caspofungin or amikacin.

### In vivo quantification of acute lung injury: alveolar-capillary barrier permeability

^125^I-albumin was injected as a vascular protein tracer and its leakage across the endothelial barrier and accumulation in the extravascular spaces of the lungs was measured using a previously described permeability index [19]. More details are provided in the Additional file 1.

### Determination of histological score

At Days 2 and 4, the lungs were removed and fixed overnight in 4% paraformaldehyde-acid and embedded in paraffin for histologic analysis. Cross-sections (3 μm thick) were stained with hematoxylin and eosin stain (Sigma-Aldrich Europe, Saint-Quentin Fallavier, France) and periodic acid Schiff. Two independent blinded investigators graded the inflammation score. The degree of peribronchial and perivascular inflammation was evaluated on a subjective scale of 0 to 3, as described elsewhere [20].

### Fluorescence staining of C. albicans in situ

Paraffin-embedded lung sections were stained with either the monoclonal antibody (mAb) 5B2 or the galenthus nivalis lectin [21,22] and examined by immunofluorescence microscopy (Leica Microsystems AG, Heerbrugg, Switzerland).

### Experimental groups

Animals were randomly assigned to the following groups: Ca: mice infected with 1 × 10^5 ^CFU of *C. albicans *at T0 and sacrificed at Day 2 or 4; Pa: mice infected with 1 × 10^7 ^CFU of *P. aeruginosa *at Day 2 and sacrificed at Day 4; CaPa: mice infected with 1 × 10^5 ^CFU of *C. albicans *at T0, infected with 1 × 10^7 ^CFU of *P. aeruginosa *at Day 2 after infection by *C. albicans*, and sacrificed at Day 4; CaPaCasp0 and CaPaCasp1: mice infected with 1 × 10^5 ^CFU of *C. albicans *at T0, treated with caspofungin from T0 or from Day 1 to Day 4, infected with 1 × 10^7 ^CFU of *P. aeruginosa *at Day 2 after infection by *C. albicans*, and sacrificed at Day 4. The experimental design is further detailed in Table 1. The sample size was four (microbial count assay), five (mortality assay), and eight animals (permeability index assay) per group. Each experiment was performed in duplicate.

**Table 1:** Experimental design of the study

	**Time(s) of infection**	**Bacterial and yeast delivery (CFU/mouse)**	**Caspofungin treatment**	**Day(s) of sacrifice**
	
**Ctr**	/	Saline solution	none	Day 2, Day 4
**Ca**	T0	1 × 10^5 ^*C. albicans*	none	Day 2, Day 4
**Pa**	Day 2	1 × 10^7 ^*P. aeruginosa*	none	Day 4
**CaPa**	T0, Day 2	T0: 1 × 10^5 ^*C. albicans* d2: 1 × 10^7 ^*P. aeruginosa*	none	Day 4
**CaPaCasp0**	T0, Day 2	T0: 1 × 10^5 ^*C. albicans* d2: 1 × 10^7 ^*P. aeruginosa*	T0 to Day 4	Day 4
**CaPaCasp1**	T0, Day 2	T0: 1 × 10^5 ^*C. albicans* d2: 1 × 10^7 ^*P. aeruginosa*	Day 1 to 4	Day 4

### Statistical analysis

Mortality rates were compared between groups by using the log rank test with Kaplan-Meier analysis. Data were analyzed by Kruskal-Wallis one-way analysis of variance test using Dunn's method to compare differences between groups (GraphPad Prism, v5.0, La Jolla, California, USA). Data are expressed as means ± standard error of the mean (SEM). *P*-values below 0.05 were considered significant.

## Results

### C. albicans tracheobronchial colonization in mice and dose-dependent pathophysiological effects

**Figure 1:**
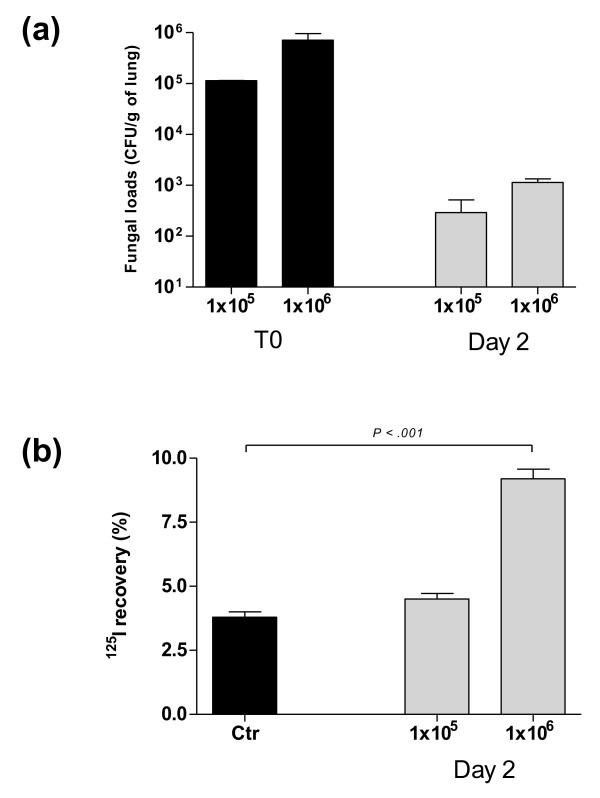
***C. albicans *tracheobronchial colonization in mice and dose-dependent pathophysiological effects**. **A**. *C. albicans *clearance from lungs. *C. albicans *loads in lungs two days after the intratracheal instillation of 1 × 10^5 ^and 1 × 10^6 ^CFU/mouse. CFU were counted on YPD plates. The data are means ± standard error (SE) (indicated by error bars). *n *= 5 mice per group. **B**. Effect of *C. albicans *on alveolar-capillary barrier permeability. Evaluation of endothelial permeability (EP) of the alveolar-capillary barrier to ^125^I-labeled bovine serum albumin two days after the intratracheal instillation of 1 × 10^5 ^and 1 × 10^6 ^CFU/mouse of *C. albicans*. The data are means ± SE (indicated by error bars). *n *= 5 mice per group.

To set up the model of tracheobronchial colonization, mice were challenged with three doses of *C. albicans *(1 × 10^5^, 1 × 10^6^, and 1 × 10^7 ^CFU per mouse). At Day 2, mortality rates were 0%, 20%, and 100% respectively, indicating a dose-dependent effect of *C. albicans*. At Day 2, after a dose of 1 × 10^5 ^and 1 × 10^6 ^CFU per mouse, the amount of live *C. albicans *in lungs was diminished by 2.5 logs for both doses (Figure 1A) and none of them induced positive fungal blood cultures (data not shown). At that time, the lung endothelial permeability was similar in the control saline solution groups and the 1 × 10^5 ^CFU group (Figure 1B). On the contrary, the efflux of the protein tracer was statistically greater for the 1 × 10^6 ^CFU group than for the control saline solution groups (*P *< 0.01). Regarding lung histopathology, an increase of inflammatory cell infiltration within bronchiole and in the surrounding lung parenchyma was observed at Day 2 in the lung of mice receiving 1 × 10^5 ^*C. albicans *cells on the photomicrographs in comparison to control mouse lungs followed by full recovery at Day 4. Immunostained lung sections from mice challenged with *C. albicans *showed the presence of *C. albicans *blastoconidia (absence of hyphae or pseudohyphae). Images of lung histopathology after *C. albicans *TBC in mice are provided in Additional file 2.

### Effect of prior C. albicans tracheobronchial colonization on P. aeruginosa pneumonia

**Figure 2:**
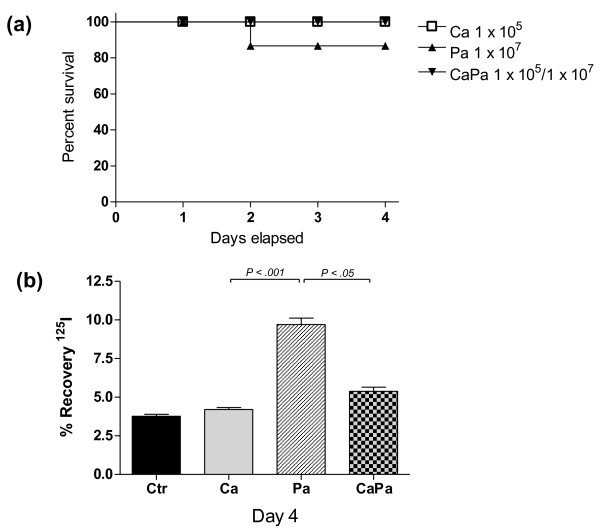
**Effect of previous *C. albicans *tracheobronchial colonization on *P. aeruginosa*-related lung injury**. **A**. BALB/c mice survival. Effect of *C. albicans *(Ca), *P. aeruginosa *(Pa), and *P. aeruginosa *after *C. albicans *(CaPa) on mouse survival during four days after intratracheal instillation of a dose of 1 × 10^5 ^CFU/mouse of *C. albicans *at T0 and of 1 × 10^7 ^CFU/mouse for *P. aeruginosa *administrated at Day 2 post-colonization. *n *= 8 mice per group. **B**. Effect of *C. albicans *and *P. aeruginosa *on alveolar-capillary barrier permeability. Evaluation of endothelial permeability (EP) of the alveolar-capillary barrier to ^125^I-labeled bovine serum albumin four days after the intratracheal instillation of a saline solution (Ctr) and in Ca, Pa, CaPa groups. The data are means ± SE (indicated by error bars). *n *= 8 mice per group.

We next addressed the issue whether prior *C. albicans *colonization in the lungs has an impact on the *P. aeruginosa *pathogenicity. When recording mortality over the course of four days post-infection, prior *C. albicans *airway colonization did not affect survival rate in case of subsequent *P. aeruginosa *infection (Figure 2A), although a trend toward an increased mortality was noted in the Pa group, but did not reach a statistical significance.

**Figure 3:**
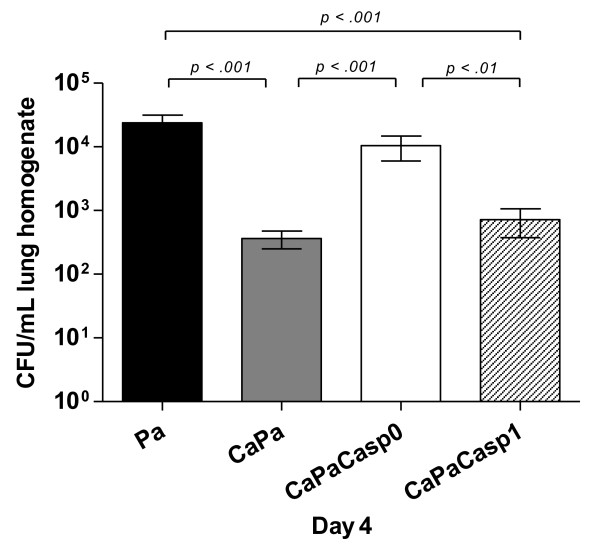
***P. aeruginosa *CFU count in lungs**. Live *P. aeruginosa *count in lung homogenates (CFU/ml) at Day 4 in Ctr, Ca, Pa, CaPa groups and caspofungin-treated group at the dose of 1 mg/kg the first day and 0.8 mg/kg the following days until Day 4, from T0 (CaPaCasp0) or from Day 1 (CaPaCasp1). The data are means ± SE (indicated by error bars). *n *= 4 mice per group.

Regarding lung endothelial permeability at Day 4, the efflux of the protein tracer in the CaPa-group was statistically greater than both the control- and Ca-groups (*P *< 0.01) but significantly lower than in the Pa-group (*P *< 0.001) (Figure 2B). In lung cultures at Day 4, the Pa-group showed a significant higher amount of live bacteria in lungs in comparison to the CaPa-group (*P *< 0.001) indicating that previous *C. albicans *airway colonization promoted the clearance of *P. aeruginosa *from the lungs (Figure 3). Also, in blood cultures at Day 4, *P. aeruginosa *detection was negative in the CaPa-group whereas they were still positive in 25% of the cases in the Pa-group (Table 2). No further *C. albicans *systemic dissemination was evidenced in the CaPa-group. The onset of *P. aeruginosa *pneumonia after *C. albicans *colonization did not affect lung *C. albicans *growth, which remained negative during the study period.

**Table 2:** Growth of *P. aeruginosa *and *C. albicans *strains in blood and effect of caspofungin

	**Bacterial growth**	**Fungal growth**
	
**Ca**	/	0/8
**Pa**	2/8*	/
**CaPa**	0/8	0/8
**CaPaCasp0**	0/8	0/8
**CaPaCasp1**	0/8	0/8

CFU were detected on BCP and YPD plates at Day 4. Positive samples are shown in the table. *CFU counts for positive samples were 20 and 40 CFU/ml. *n *= 4 mice per group.

**Figure 4:**
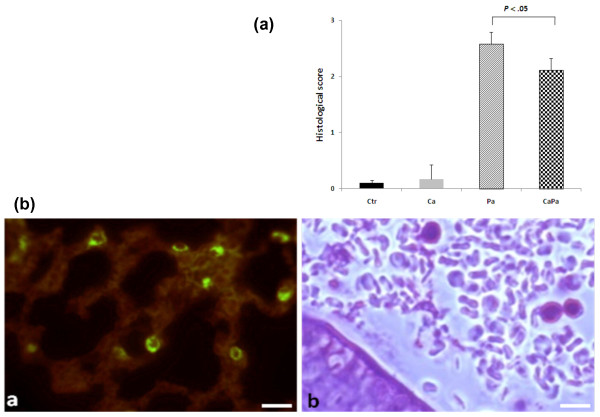
**Lung histopathology after sequential infection with *C. albicans *and *P. aeruginosa *in mice**. **A**. Histological score of lung sections from BALB/c mice on Day 4. Peribranchiol and perivascular lung inflammation in mice was measured by two independent blinded examiners. Data are expressed as mean ±SE for each group. *P *< 0.05 for CaPa *vs *Pa mice. **B**. Immunofluorescence and periodic acid Schiff staining for *C. albicans *localization in lungs of BALB/c mice on Day 4. (a) Representative section of lung from mice challenged with both *C. albicans *and *P. aeruginosa *stained with fluorescent galenthus nivalis lectin (GNL) specific for terminal α-D-mannosyl, preferentially α-1,3 residues of *C. albicans*. The scale bars represent 10 μm. (b) Lung section from mouse receiving *C. albicans *and *P. aeruginosae *stained with PAS (periodic acid Schiff). The scale bars represent 5 μm.

An important inflammatory cell infiltration within bronchiole and in the surrounding lung parenchyma was observed in mice receiving *P. aeruginosa *as evidenced by the histological score of lung sections (Figure 4A). Conversely, the CaPa-group had a significantly lower score of pathological lesions than the Pa-group on the histological score of lung sections (*P *< 0.05) (Figure 4A). Lung immunostaining at Day 4 showed the presence of *C. albicans *blastoconidia exclusively (Figure 4C-Images a and b). Together, our results suggest that *C. albicans *colonization prior to *P. aeruginosa *infection decreases the *P. aeruginosa *bacterial load and minimizes the lung lesions.

### Effect of antifungal treatment on C. albicans interference with P. aeruginosa pneumonia

**Figure 5:**
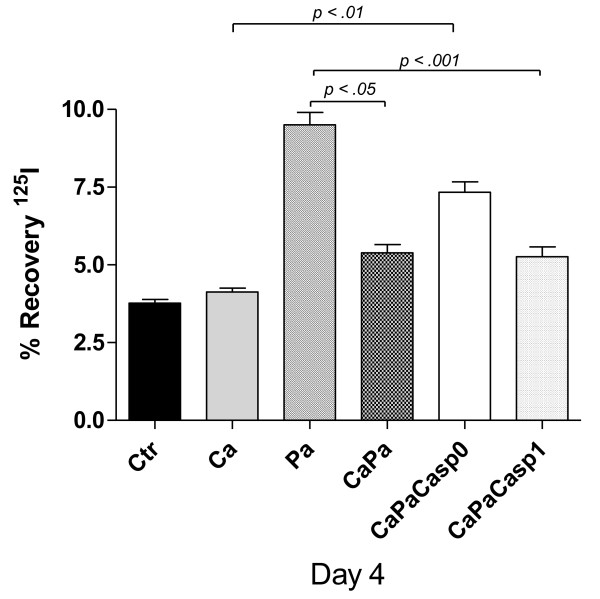
**Effect of caspofungin on *alveolar-capillary barrier permeability***. Evaluation of endothelial permeability (EP) of the alveolar-capillary barrier to ^125^I-labeled bovine serum albumin at Day 4 in Ctr, Ca, Pa, CaPa groups and caspofungin-treated group at the dose of 1 mg/kg the first day and 0.8 mg/kg the following days until Day 4, from T0 (CaPaCasp0) or from Day 1 (CaPaCasp1). The data are means ± SE (indicated by error bars). *n *= 8 mice per group.

Antifungal caspofungin has been used to test whether it might reverse the effect of *C. albicans *airway colonization on subsequent *P. aeruginosa *pneumonia. The treatment was initiated upon infection at T0 or after a delay at Day 1 post-colonization. The fungicidal effect of caspofungin in mouse lungs was previously assessed at Day 2 and no positive fungal cultures were collected in both the CaCasp0- and the CaCasp1-groups (data not shown). Furthermore, the lack of impact of caspofungin on endothelial permeability was also confirmed at Day 1 after a single intraperitoneal injection of 1 mg/kg of caspofungin in control saline solution-instilled mice (data not shown). Caspofungin administration from T0 reversed the effect of previous airway colonization by *C. albicans *on *P. aeruginosa*-induced lung injury (Figure 5). Thus, when the antifungal is administered early the lung injury induced by *P. aeruginosa *persists and the endothelial permeability showed by means of protein tracer leak is enhanced to the level of the Pa-group. In contrast, caspofungin administration from Day 1 did not reverse the *C. albicans *effect as an equal amount of protein efflux tracer was observed in the Ca- and CaPa-groups and was still significantly different from the Pa-group (*P *< 0.001).

Regarding lung bacterial counts at Day 4, the effect of caspofungin was different depending on the time of administration (Figure 3). Administration from T0 (the CaPaCasp0-group) significantly abolished the decrease of positive specimens observed in the CaPa-group (*P *< 0.001). Conversely, delayed administration from day 1 (CaPaCasp1-group) resulted in collecting a roughly similar amount of live *P. aeruginosa *in lungs than in the CaPa-group creating a significant difference with the CaPaCasp0-group (*P *< 0.01).

## Discussion

The present study was designed to determine the contribution of *C. albicans *airway colonization to *P. aeruginosa *pathogenicity in immunocompetent mice. Our results indicate that prior short-term *C. albicans *airway colonization reduced *P. aeruginosa*-induced lung injury and the amount of live *P. aeruginosa *in lungs. This effect is reversed by fungicidal drug caspofungin when initiated concomitantly to *C. albicans *infection.

The prerequisite to the study was the set-up of tracheobronchial colonization by *C. albicans *according to the definition of colonization, which is the presence of a pathogen that does not cause damages on the lung parenchyma. The dose of 1 × 10^5 ^CFU of *C. albicans *per mouse matched with this criterion as no invasive disease occurred. After *P. aeruginosa *infection, a trend toward a higher survival rate in the *C. albicans*-colonized mice was observed. This result is consistent with data comparing groups of mice instilled simultaneously with *P. aeruginosa *and *C. albicans *or with *P. aeruginosa *alone showing a significant difference of survival in favor of the *C. albicans*-colonized group at Day 7 [23]. Then, it was found that previous *C. albicans *airway colonization was associated with an increase in lung *P. aeruginosa *clearance compared to the non-colonized-group. These data differ from the previous study, which did not detect a significant decrease in quantitative bacterial burden in the group receiving simultaneous administration of *C. albicans *along with *P. aeruginosa *[23]. However, a major difference is that bacterial loads were recorded early after the co-infection between 3 and 20 h. Another study, which addressed the issue of prevalence of *P. aeruginosa *pneumonia in rats colonized by *C. albicans*, evaluated the quantitative bacterial cultures of *P. aeruginosa *in rat lungs at 48 h post-infection [16]. Subsequent to *C. albicans *colonization obtained by intratracheal instillation (2 × 10^6 ^CFU per rat three days in a row), a low dose of *P. aeruginosa *(1 × 10^4 ^CFU per rat) was delivered at Day 2 post-colonization. The bacterial burden was significantly higher at 48 h in rats instilled with *C. albicans *before *P. aeruginosa *compared to rats instilled with saline solution or ethanol-killed *C. albicans *before *P. aeruginosa*. Contrary, in our experimental model, *P. aeruginosa *dissemination in the bloodstream showed a trend toward a decrease in the case of prior *C. albicans *colonization. Although bacterial dissemination is multifactorial depending on the magnitude of alveolar-capillary barrier injury [24] as well as the strain virulence and the size of the inoculum [25], the decrease was most likely due to the decrease of alveolar-capillary barrier injury since the *P. aeruginosa *strain used and the size of the inoculum administrated were identical in both groups. Regarding histolopathologic results, the inflammation score decreased in the case of previous *C. albicans *airway colonization in comparison to *P. aeruginosa *infection alone suggesting that primary immune activation could reduce *P. aeruginosa *pathogenicity. This observation was consistent with a decrease in *P. aeruginosa *lung loads and a decrease in the lung permeability index in the CaPa group in comparison to the Pa group. These results differ from a study previously mentioned which concluded that previous *C. albicans *colonization lowered the threshold of *P. aeruginosa *load necessary to induce parenchymal injury since in rats given *C. albicans*, histologic aspect of *P. aeruginosa *pneumonia was significantly more frequent than in controls or ethanol-killed *C. albicans *rats [16]. Overall, the results may differ between mice and rats, and between different strains of *P. aeruginosa *owing to differential susceptibility to pneumonia.

In the second part of this study, the influence of fungicidal caspofungin was tested. The use of caspofungin aimed at detecting a difference between colonization with live or killed *C. albicans*. For that purpose, two target times for treatment initiation were chosen, from T0 or from Day 1. Regarding the alveolar-capillary barrier injury, the use of caspofungin resulted in distinct effects: reversal of the decrease in the protein tracer leakage when initiated at T0 or maintenance of the decrease in the protein tracer leakage when initiated at Day 1. The difference observed between the T0- and the Day 1-treated group suggests that the viability and/or the growth of *C. albicans *makes a difference in reducing the magnitude of alveolar-capillary barrier injury.

Overall, these results raise three hypotheses: first, a competitive effect regarding the adhesion of the pathogens to lung epithelial surface. Indeed, they both use ligands to recognize the glycoconjugates at the surface of epithelial cells [26,27]. Recently, it has been demonstrated that *P. aeruginosa *lectins LecA and LecB, which are involved in adhesion to epithelial cells, contribute to *P. aeruginosa*-induced lung injury [17]. The neutralization of these lectins by the administration of specific lectin inhibitors was remarkably effective in improving lung injury. *C. albicans *adherence to host tissue is controlled by the ALS (agglutinin-like sequence) gene family which encodes a group of glycosyl-phosphatidyl-inositol (GPI)-linked cell surface proteins that function as adhesins that bind to the cell surface [28]. The second hypothesis is a bactericidal effect mediated by higher-inducible lung mucosal innate response by live *C. albicans*. This hypothesis is supported by the decrease in inflammation score in case of previous *C. albicans *airway colonization in comparison to *P. aeruginosa *infection alone, and by the fact that T0 caspofungin treatment resulted in a higher rate of bacterial growth. Finally, *C. albicans *produces farnesol, a cell-to-cell signaling molecule that could act as a quorum-sensing antagonist of *P. aeruginosa *[14,29]. The addition of farnesol to cultures of *P. aeruginosa *leads to decreased production of the *Pseudomonas *quinolone signal (PQS) and the PQS-controlled downstream virulence factor, pyocyanin [30]. Furthermore, it has been shown that the *C. albicans *farnesol has also the ability to inhibit swarming motility in *P. aeruginosa *cystic fibrosis clinical isolates [31]. All together, the reduction in PQS-pyocyanin production and swarming mobility may also have implications for the interaction between *P. aeruginosa *and the host.

The present study has several limitations that prevent extrapolating the results to the chronically colonized and/or critically ill patients at risk for VAP. First, the short term *C. albicans *colonization in the model does not correctly reflect the situation of these patients. Indeed, the amount of *C. albicans *in lungs cannot be substantially sustained over time in immunocompetent BALB/c mice, as already described elsewhere [32]. Consequently, *P. aeruginosa *pneumonia had to be generated only 48 hours after the prior fungal colonization. The addition of a control experimental group testing the impact of killed *C. albicans *would have been of interest to assess the need of live *C. albicans *to produce the effects described. Concern can also be raised regarding some *in vitro *data indicating a decrease of *P. aeruginosa *growth following exposure to halogenated anesthetics [33], although it occurred after several hours of exposure and has not been investigated *in vivo*. The short duration of mutual contact and interaction of *C. albicans *and *P. aeruginosa *in the airways (48 h) represents another potential bias of the present study. Furthermore, a dose/effect study testing various doses of *P. aeruginosa *to generate pneumonia could better document the *in vivo *dynamics of bacterial-fungal interactions. Performing microbial CFU counts in spleen and liver could better assess microbial dissemination. Finally, this relationship is studied in normal lungs and in the absence of any airway prosthetic device, which largely promotes microbial community networking [12].

## Conclusions

The present results demonstrate that *P. aeruginosa*-related lung injury is reduced when preceded by short term *C. albicans *airway colonization. Regarding the use of the antifungal drug caspofungin, reduced *P. aeruginosa*-related lung injury is reversed when the treatment is initiated at T0, and maintained when the treatment is started one day after the onset of *C. albicans *colonization. The study illustrates the complex relationships between fungi and bacteria consistently with a number of other works in which cross-kingdom interactions result in very different effects (from synergy to antagonism). Additionally, the impact of antifungal agents on the fungal-bacterial ecosystem is poorly understood. Further *in vitro *and *in vivo *studies are required using cell wall *C. albicans *extracts such as glucans or mannans during *P. aeruginosa *infection in order to better understand the molecular mechanisms involved.

## Key messages

• In this study, murine *P. aeruginosa*-induced lung injury measured at 48 h post-infection is reduced when preceded by short term *C. albicans *airway colonization.

• Additionally, short-term *C. albicans *colonization results in a reduction of the amount of *P. aeruginosa *in murine lungs at 48 h post-infection.

• Using the fungicidal drug caspofungin upon *C. albicans *colonization reverses these effects.

## Abbreviations

ALS: agglutinin-like sequence; BCP: bromocresol purple; Ca: *Candida albicans*; CaPaCasp: *Candida albicans*, *Pseudomonas aeruginosa*, Caspofungin; CFU: colony-forming units; GPI: glycosyl-phosphatidyl-inositol; Pa: *Pseudomonas aeruginosa; *PQS: *Pseudomonas *quinolone signal; SDA: Sabouraud dextrose agar; TBC: Tracheobronchial colonization; VAP: ventilator-acquired pneumonia; YPD: yeast peptone dextrose.

## Competing interests

The authors declare that they have no competing interests.

## Authors' contributions

FA participated in the design of the study, carried out the *in vivo *experiments, performed the statistical analysis and drafted the manuscript. SJ carried out the histological and immunofluorescence assays and helped to draft the manuscript. SN, BS KF, FV, CC, DP and BG participated in the design and coordination of the study and helped to draft the manuscript. All authors read and approved the final manuscript.

## Supplementary Material

Additional file 1 ***In vivo *quantification of acute lung injury: alveolar-capillary barrier permeability**. Method of measurement of alveolar-capillary barrier permeability. Click here for file

Additional file 2 **Lung histopathology after *C. albicans *tracheobronchial colonization in mice**. Supplemental figures of lung histopathology at Day 2 post-infection with *C. albicans* Click here for file
