# Supplementary material for: Short term Candida albicans colonization reduces Pseudomonas aeruginosa-related lung injury and bacterial burden in a murine model
Source: Crit Care. 2011 Jun 20;15(3):R150. doi: 10.1186/cc10276 (PMC3219024; doi:10.1186/cc10276)
Supplement: Additional file 1 — In vivo quantification of acute lung injury: alveolar-capillary barrier permeability. Method of measurement of alveolar-capillary barrier permeability. [file cc10276-S1.PDF]

Online Supplemental Material

Short term *Candida albicans* colonization reduces *Pseudomonas aeruginosa* load and lung injury in a mouse model

Florence Ader, Samir Jawhara, Saad Nseir, Boualem Sendid, Karine Faure, Fanny Vuotto, Chanez Chemani, Daniel Poulain, and Benoit Guery

Supplemental Materials and Methods

In vivo quantification of acute lung injury: alveolar-capillary barrier permeability

0.5 ml of ^{125}I-labeled bovine serum albumin (1 µCi) (HAS; CIS Biointernational, Gif-sur-Yvette, France) was injected intraperitoneally 2 h before intraperitoneal injection of pentobarbital sodium (Sanofi, Libourne, France), followed by sternotomy, exsanguination and lungs removal. Radioactivity and hemoglobin (Hb) concentration were measured in blood. The lungs were weighed and radioactivity was counted prior to homogenization and centrifugation (Polytron, PT 1600E; Fischer Bioblock Scientific, Switzerland). The Hb content of the supernatant was also measured. The PI used to express the permeability of the alveolar-capillary membrane was calculated as follows: PI = {[Radioactivity count for lungs – (Radioactivity count for intravascular blood per gram of blood x Q_B)]/(Radioactivity count for intravascular blood per gram of blood x Weight of mouse)} x 100, where Q_B is the weight of intrapulmonary blood and was calculated as follows: Q_B = (Weight of lung plus water x Hb concentration in supernatant x water ratio for homogenate x 1.039)/(Hb concentration in blood x water ratio for blood).
