# Supplementary material for: Short term Candida albicans colonization reduces Pseudomonas aeruginosa-related lung injury and bacterial burden in a murine model
Source: Crit Care. 2011 Jun 20;15(3):R150. doi: 10.1186/cc10276 (PMC3219024; doi:10.1186/cc10276)

Supplemental Figure

Lung histopathology after *C. albicans* tracheobronchial colonization in mice

Image below: representative photomicrographs of lung sections on day 2. Lung tissue was fixed, sectioned at 3 μm thickness and stained with hematoxylin and eosin. (a) Lung section from control mouse receiving PBS. (b) Lung section from mouse inoculated with 1 x 10^5 live *C. albicans* cells by intratracheal instillation. The scale bars represent 50 μm. (c and d) Representative photomicrographs of lung sections on day 4. (c) Lung section from control mouse receiving PBS. (d) Lung section from mouse inoculated with 1 x 10^5 live *C. albicans* cells by intratracheal instillation.

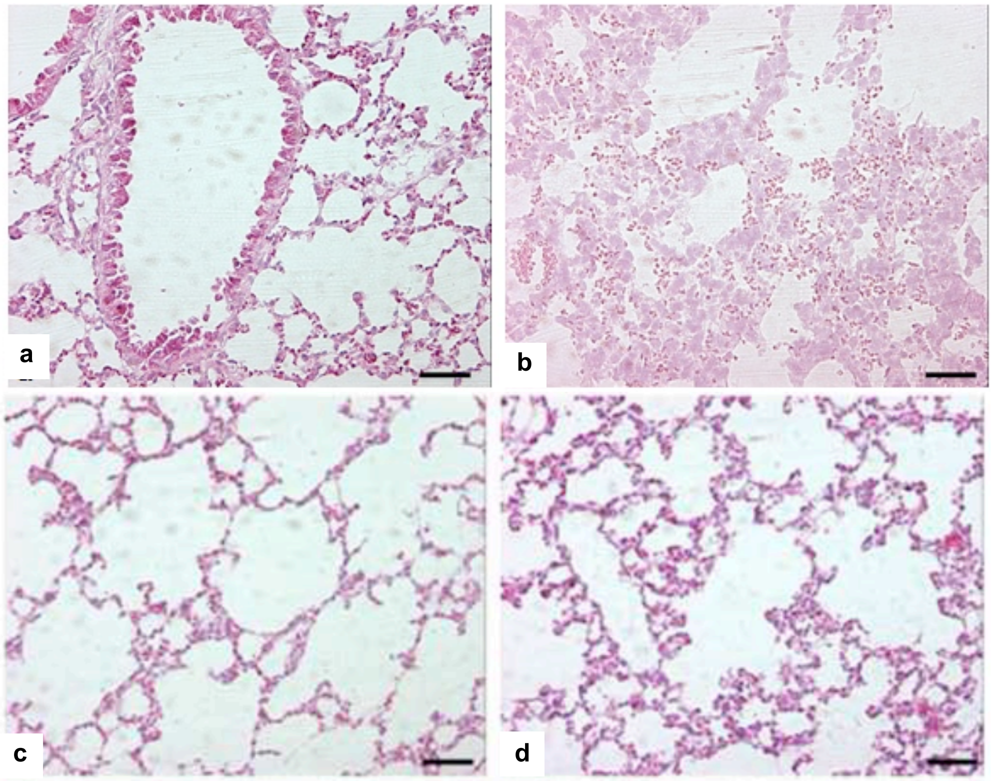

Image below: immunofluorescence and periodic acid Schiff staining for *C. albicans* localization in lungs of BALB/c mice on day 2 post-*C. albicans* challenge. (a) Representative section of lung from mice challenged with *C. albicans* alone immuno-stained with an anti-β-1,2-linked oligomannose monoclonal antibody showing *C. albicans* blastoconidia in the lung. The scale bars represent 50 μm. (b) Boxed region in image A is shown at higher magnification. The scale bars represent 5 μm.

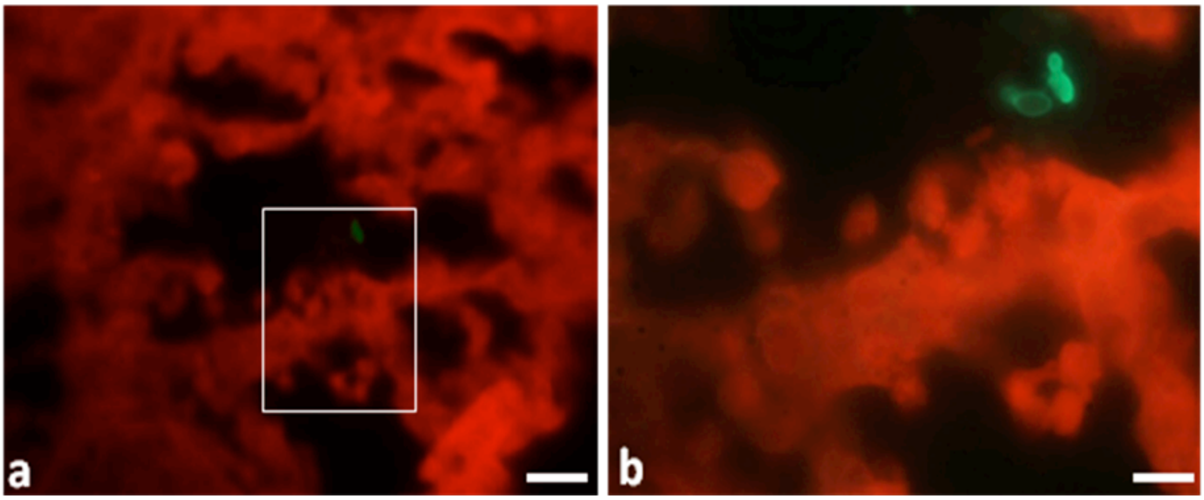

Supplement: Additional file 2 — Lung histopathology after C. albicans tracheobronchial colonization in mice. Supplemental figures of lung histopathology at Day 2 post-infection with C. albicans [file cc10276-S2.PDF]
